# Iron deposition is associated with motor and non-motor network breakdown in parkinsonism

**DOI:** 10.3389/fnagi.2024.1518155

**Published:** 2025-01-20

**Authors:** Fangda Leng, Yue Gao, Fan Li, Luhua Wei, Yunchuang Sun, Fang Liu, Ying Zhu, Jianxing Qiu, Zhaoxia Wang, Yiwei Zhang

**Affiliations:** ^1^Department of Neurology, Peking University First Hospital, Beijing, China; ^2^Department of Radiology, Peking University First Hospital, Beijing, China; ^3^Department of Neurology, Tsinghua University First Hospital, Beijing, China

**Keywords:** Parkinson’s disease, multiple system atrophy, progressive supranuclear palsy, iron deposition, brain network

## Abstract

**Background:**

Iron deposition has been observed in Parkinsonism and is emerging as a diagnostic marker for movement disorders. Brain functional network disruption has also been detected in parkinsonism, and is believed to be accountable for specific symptoms in parkinsonism. However, how iron deposition influences brain network remains to be elucidated.

**Methods:**

We recruited 16 Parkinson’s disease (PD), 8 multiple system atrophy (MSA) and 7 progressive supranuclear palsy (PSP) patients. T1-weighted, susceptibility weighted images and resting-state functional MRI (rs-fMRI) were acquired. Quantitative susceptibility mapping (QSM) analysis was performed to quantify iron deposition in substantia nigra, putamen and dentate nucleus. Cerebellar network, sensorimotor network, default mode network and language networks were segregated using independent analysis. Network and iron deposition status were evaluated in relation to diagnostic groups, motor and non-motor symptoms. The relationship between quantitative iron deposition and brain network status was further interrogated. To further validate the findings, 13 healthy controls and 37 PD patients who had available T1 and rs-fMRI scans were selected from Parkinson’s progression markers initiative (PPMI) database, and network analysis was performed.

**Results:**

In local cohort, compared to PD, MSA patients showed greater iron deposition in putamen, while PSP patients had greater iron deposition in caudate nucleus and thalamus. Cerebellar and language networks showed significant difference across diagnostic groups, while default mode network and sensorimotor network did not. MSA patients had significantly impaired cerebellar network and language networks compared to PD patients. Cerebellar network was positively associated with motor symptom scores while language network was positively associated with MoCA scores in the patients. Iron deposition was negatively associated with both networks’ activity in the patients. In PPMI cohort, impairment was found in both cerebellar and language networks in PD. Cerebellar and language networks correlated with motor and cognitive impairment, respectively.

**Conclusion:**

Cerebellar network and language networks are differently influenced in MSA, PD and PSP, which can serve as potential diagnostic marker. Impairment of cerebellar network and language network are associated with motor symptoms and cognitive impairment, respectively. Moreover, dysfunction of the networks is associated with iron deposition in deep nuclei (SN, DN, Putamen).

## 1 Introduction

Parkinsonism is characterized by bradykinesia, rest tremor, rigidity, and postural instability (Postuma et al., 2015). Parkinson’s disease (PD) is the most common disorder that causes parkinsonism, while multiple system atrophy with predominant parkinsonism (MSA-p) and progressive supranuclear palsy (PSP) are among the differential diagnosis of PD (Hoglinger et al., 2017; Wenning et al., 2022). While the latter disorders are accompanied by distinctive symptoms such as dysautonomia and ophthalmoparesis, differential diagnosis at early stages can be challenging. Apart from parkinsonian symptoms, these neurodegenerative disorders are often accompanied by cognitive dysfunction involving language, executive and social cognitive domains. Cognitive impairment is believed to be more prevalent among PSP and PD patients, with MSA patients being relatively less involved (Poletti et al., 2012; Raimo et al., 2023).

The clinical challenge of differential diagnosis has called for novel biomarkers, among which imaging markers take crucial part (Mitchell et al., 2021; Saeed et al., 2020). Most used imaging markers are structural measurements that reflect disease specific anatomical changes, such as midbrain atrophy in PSP and putamen atrophy in MSA (Quattrone et al., 2018; Brooks et al., 2009). While those structural markers have shown satisfactory specificity, their limited sensitivity presses for markers of other imaging modalities that offer better early-stage sensitivity (Saeed et al., 2020). As movement disorders usually involve iron deposition in disease-specific anatomical location, susceptibility weighted imaging (SWI) has emerged as a promising marker (Gupta et al., 2010). As an example, loss of swallow-tail sign in SWI images reflects iron deposition in substantia nigra, and is a reliable marker of PD (Brammerloh et al., 2022). Iron deposition in putamen and cerebellar nuclei has been observed in MSA (Mohammadi and Ghaderi, 2024), whereas in PSP patients, iron deposition is readily observed in subthalamic nucleus, red nucleus and globus pallidum (Gupta et al., 2010; Sjostrom et al., 2017). Recent studies have also suggested association between iron deposition and motor symptoms in movement disorders (Zeng et al., 2024).

Alongside SWI, functional imaging is increasingly recognized as a potential biomarker in movement disorders. It is believed that distinct brain network connectivity patterns reflect disease specific changes and network information could effectively support differential diagnosis of Parkinsonian syndromes (Spetsieris et al., 2009; Burciu et al., 2015). Indeed, the brain functions as a network organ and it is widely speculated that neurological manifestations are rooted in network dysfunction of the brain. For instance, in Parkinson’s disease, electrophysiological studies have demonstrated bradykinesia and tremor to be related to deranged subthalamic nucleus—motor cortex connection, and cerebello-thalamo-cortical motor loop, respectively (Lee et al., 2021; Dirkx and Bologna, 2022). And it is reasonable to hypothesize that same phenomenon could be detected by fMRI methods, albeit with lower temporal resolution, and that non-motor symptoms also root in certain network changes. Moreover, we posit that iron deposition could cause local and distal brain network dysfunction, linking the local neurological abnormality to clinical manifestation.

## 2 Methods

### 2.1 Patient recruitment and inclusion criteria

Patients were recruited from Peking University First Hospital from November 2021 to September 2023. The diagnosis of Parkinson’s disease, multiple system atrophy, progressive supranuclear palsy were made according to the Movement Disorder Society criteria for PD (Postuma et al., 2015), MSA (Wenning et al., 2022) and PSP (Hoglinger et al., 2017), respectively. The clinical diagnosis of parkinsonism was made based on clinical presentation, and an initial diagnosis of PD, MSA or PSP were made by at least 2 experienced neurologists in agreement in outpatient settings. Clinical diagnoses were reconfirmed after inpatient assessment and expert panel review of the cases. Exclusion criteria include: (1) major psychiatric disorders including schizophrenia, major depressive disorder, schizoaffective disorders; (2) Significant CNS disorders including debilitating stroke, imaging evidence of excessive cerebral small vessel disease (Fazekas grade 3), CNS demyelination disorders; (3) safety concerns for MRI compatibility, and inability to withstand the MRI scan (e.g., specific phobia of enclosed space); (4) History of intracranial surgeries, including deep brain stimulation; (5) red flags in the diagnosis of respective diseases, (6) overt dementia as evidenced by clinical dementia rating ≥ 2.

The study is approved by institutional review board of Peking University First Hospital (IRB00001052-17043). The studies were conducted in accordance with the local legislation and institutional requirements. Written informed consent was obtained from all participants.

To validate finding from our local cohort, we also selected 13 health control (HC) and 37 PD patients from Parkinson’s progression markers initiative (PPMI) database. The selection criteria were: (1) Explicitly assigned to HC or PD category by PPMI study institutes; (2) Had complete T1 weighted MRI, rs-fMRI, MoCA test and UPDRS motor test data acquired within 6 months of whichever first assessment. Detailed clinical protocol of PPMI study can be found at https://www.ppmi-info.org/sites/default/files/docs/002_Protocol_AM4_v3.0_23July2024_Final.pdf.

### 2.2 Clinical assessment

After informed consent, participants were invited to have inpatient assessments. Demographic information, disease duration, and levodopa equivalent daily dose (LEDD) were recorded. Unified movement disorder assessments included Unified Parkinson’s Disease Rating Scale (UPDRS) for PD patients, Unified Multiple System Atrophy Rating Scale (UMSARS) for MSA patients, and Progressive Supranuclear Palsy Rating Scale (PSPRS) for PSP patients. Motor assessments were performed in ‘on’ medication states. Cognitive performance was assessed with Montreal Cognitive Assessment (MoCA). Other evaluations included autonomic function assessments, electronystagmography, and levodopa challenge test. Patients had out-patient follow up for at least 1 year after discharge and all diagnoses were reviewed before analysis.

### 2.3 Image acquisition

For local patients, images were acquired in ‘on’ medication sate using a 3 T scanner (Ingenia 3.0 T; Philips Healthcare, Best, Netherlands). Structural MRI data were acquired using a 3D T1-weighted imaging. Turbo field echo compressed SENSE (TFE-CS) sequence on Philips unit with the following parameters: repetition time (TR) = 6.5 ms; echo time (TE) = 2.9 ms; flip angle: 8°; FOV: 240 × 240 mm; and voxel size: 1.0 × 1.0 × 1.0 mm.

Resting-state fMRI was acquired using an echo-planar imaging (EPI) sequence with the following parameters: TR = 2,970 ms, TE = 30 ms, voxel size: 2.0× 2.0× 3.0 mm^3^, 3 mm slice gap, and flip angle (FA) 90°. Before resting-state data acquisition, we instructed participants to close their eyes, relax, and not engage in any particular mental activity during the scan.

A multi-echo gradient-echo (GRE) sequence was used for SWI-plus data acquisition with the following parameters: TR = 41 ms, five TEs =7.5/15/22.5/30/37.5 ms, FA = 15°, voxel size = 0.67 × 0.67× 2.0mm^3^, and bandwidth = 217 Hz/px, number of slices: 66, slice orientation: F-H.

PPMI imaging data selected in the current analysis was acquired with SIEMENS prisma 3 T scanner. Detailed parameters can be found in https://www.ppmi-info.org/sites/default/files/docs/PPMI2.0_002_MRI_TOM_Final_v3.0_20210727_FE.pdf.

### 2.4 Image processing

Analyses of fMRI data were performed using CONN toolbox release 22.7 (Whitfield-Gabrieli and Nieto-Castanon, 2012). fMRI volumes were realigned using SPM realign & unwarp function, where all scans were coregistered to the first volume using a least squares approach and a rigid body transformation, then resampled using b-spline interpolation for motion correction. Temporal misalignment between different slices was corrected using since temporal interpolation. Potential outlier scans were identified using ART as acquisitions with framewise displacement above 0.5 mm or global BOLD signal changes above 3 standard deviations. Reference functional image was computed by averaging all fMRI volumes for each participant. Functional and anatomical data were normalized into standard MNI space, segmented and resampled to 2 mm isotropic voxels following a direct normalization procedure using SPM unified segmentation and normalization algorithm. Lastly, functional data were smoothed using spatial convolution with a 6 mm Gaussian kernel.

fMRI data were denoised using a standard denoising pipeline including the regression of potential confounding effects characterized by white matter timeseries, CSF timeseries, motion parameters and their first order derivatives, outlier scans, followed by bandpass filtering between 0.008 Hz and 0.09 Hz.

A singular value decomposition (SVD) with 64 components was used as a subject-specific dimensionality reduction. Independent component analyses were performed to estimate 40 temporally coherent networks from the fMRI data combined across all subjects. The dimensionality of the concatenated data was further reduced using SVD with 40 components, and fast-ICA was used to identify spatially independent group-level networks from the resulting components. Last, GICA3 back-projection was used to compute ICA maps associated with these same networks separately for each individual subject.

The spatial properties of ICA networks were compared against established functional networks, and dice index was used to determine the corresponding functional networks of ICA components (Supplementary Figure 1). As illustrated by previous efforts, cerebellar network, sensorimotor network, default mode network and language networks were selected *a priori* as networks of interest (Supplementary Figure 2).

SWI images were processed using JHU/KKI QSM toolbox (Li et al., 2019). Laplacian phase unwrapping was first performed followed by background removal using variable-kernel sophisticated harmonic artifact reduction for phase data (V-SHARP) method, with the average image of all echoes as reference (Fang et al., 2017). Images were then smoothed with an 8 mm Gaussian Kernel. Quantitative susceptibility was calculated using thresholded k-space division (TKD) method. SWI images were then coregistered with T1-weighted images normalized into standard MNI space, segmented and resampled to 2 mm isotropic voxels following direct normalization. Magnetic susceptibility value (measured in ppm) was sampled in caudate nuclei, putamen, substantia nigra and dentate nuclei.

### 2.5 Statistical analysis

Statistical analyses were performed with R (4.4.1) and SPM12 for clinical variables and parametric images, respectively. Kruskal Wallis test was performed for cross-group comparison, Mann Whitney *U* test was used for group-wise comparison of continuous variables, and Chi-square test was applied to categorical variables.

Group-level analyses of ICA maps were performed using General Linear Models (GLM). For each ICA component (network of interest), ANOVA was performed to determine if any difference existed across diagnostic groups. For those networks with positive *F*-test (namely cerebellar network and language network), *post-hoc t*-tests were performed to establish unique network changes specific to the diagnostic group. Regression analyses were further performed on brain network and susceptibility maps to interrogate the association between clinical assessments (motor performance and MoCA scores) and brain network activity or with iron deposition. Inferences were performed at the level of individual clusters (groups of contiguous voxels). Sex and disease duration were corrected in the regression models and group comparisons. Cluster-level inferences were based on parametric statistics from Gaussian Random Field theory. Results were thresholded using a combination of a cluster-forming *p* < 0.005 voxel-level threshold, and a family-wise corrected p-FDR < 0.05 cluster-size threshold. An illustration of analysis framework can be found in Supplementary Figure 3.

### 2.6 Data availability

Local study data can be shared upon reasonable request to the corresponding authors. PPMI data used in the preparation of this article was obtained on [2024-11-25] from the Parkinson’s Progression Markers Initiative (PPMI) database (https://www.ppmi-info.org/access-data-specimens/download-data), RRID:SCR_006431. For up-to-date information on the study, visit https://www.ppmi-info.org.

## 3 Results

### 3.1 Demographic and clinical features of participants

16 PD patients, 8 MSA patients, 7 PSP patients were recruited in our local cohort, and a further 13 HC and 37 PD patients were selected form PPMI database. Sex distribution, age and disease duration did not differ significantly across the groups. In our local cohort, the median of MoCA scores were 24.5, 26 and 22 for PD, MSA and PSP patients, respectively. Median UPDRS motor score was 26.5 for PD patients, median UMSARS score was 19.5 and median PSPRS motor section score was 20. Demographic and clinical profile of study cohort is summarized in Table 1 and extended clinical information can be found in Supplementary Table 1.

**Table 1:** Demographic information of study participants.

	PD (local)	MSA	PSP	*p*	PD (PPMI)	HC (PPMI)	*p*
N	16	8	7	-	13	37	
Male	7	5	5	0.29	9	23	0.64
Female	9	3	2		4	14	
Age	61.5 (56, 72)	65.5 (59, 70)	72 (68, 76)	0.08	65 (59, 74.5)	66 (63, 74.5)	0.84
Disease duration	2.8 (1.8, 5.0)	1.25 (0.9, 2.2)	1 (1, 2)	0.052	-	-	
MoCA	24.5 (22, 26)	26 (23, 27)	22 (20, 23)	0.08	29 (28, 29)	27 (26, 29)	0.077
UPDRSm	26.5 (21, 36)	-	-	-	23 (16, 30)	0 (0, 2)	<0.001
UMSARSm	-	19.5 (12, 27)	-	-			
PSPRSm	-	-	20 (14, 29)	-			

Data were presented as median (1st quartile, 3rd quartile). *p*, *p*-values of Kruskal Wallis or Chi-square tests.

### 3.2 Comparison of iron deposition in different diagnostic groups

**Figure 1:**
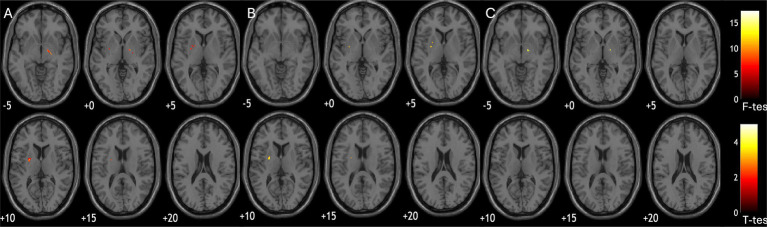
Group comparisons of susceptibility parametric maps. **(A)** significant difference of susceptibility across diagnostic groups (ANOVA); **(B)** MSA patients had more iron deposition in putamen compared to PD; **(C)** PSP patients had more iron deposition in pallidum compared to PD.

Quantitative susceptibility differed in putamen across groups, but did not survive FDR correction (*F* = 4.7, *p* = 0.17, *p*-adj = 0.17). Voxel-wise ANOVA suggested cross-group difference in left putamen [(−24, −3, −9); cluster size = 433; cluster-p-FDR < 0.001] and right globus pallidus [(17, −7, −2); cluster size = 181; cluster-p-FDR = 0.008, Figure 1A]. Post-hoc group wise comparisons showed MSA patients had increased iron deposition in left putamen compared to PD patients [(−24, −3, 9); cluster size = 374; cluster-p-FDR < 0.001, Figure 1B]; while PSP patients had increased iron deposition in right pallidum compared to PD patients [(17, −7, 2); cluster size = 186, cluster-p-FDR = 0.04, Figure 1C].

### 3.3 Comparison of functional networks in different diagnostic groups

**Figure 2:**
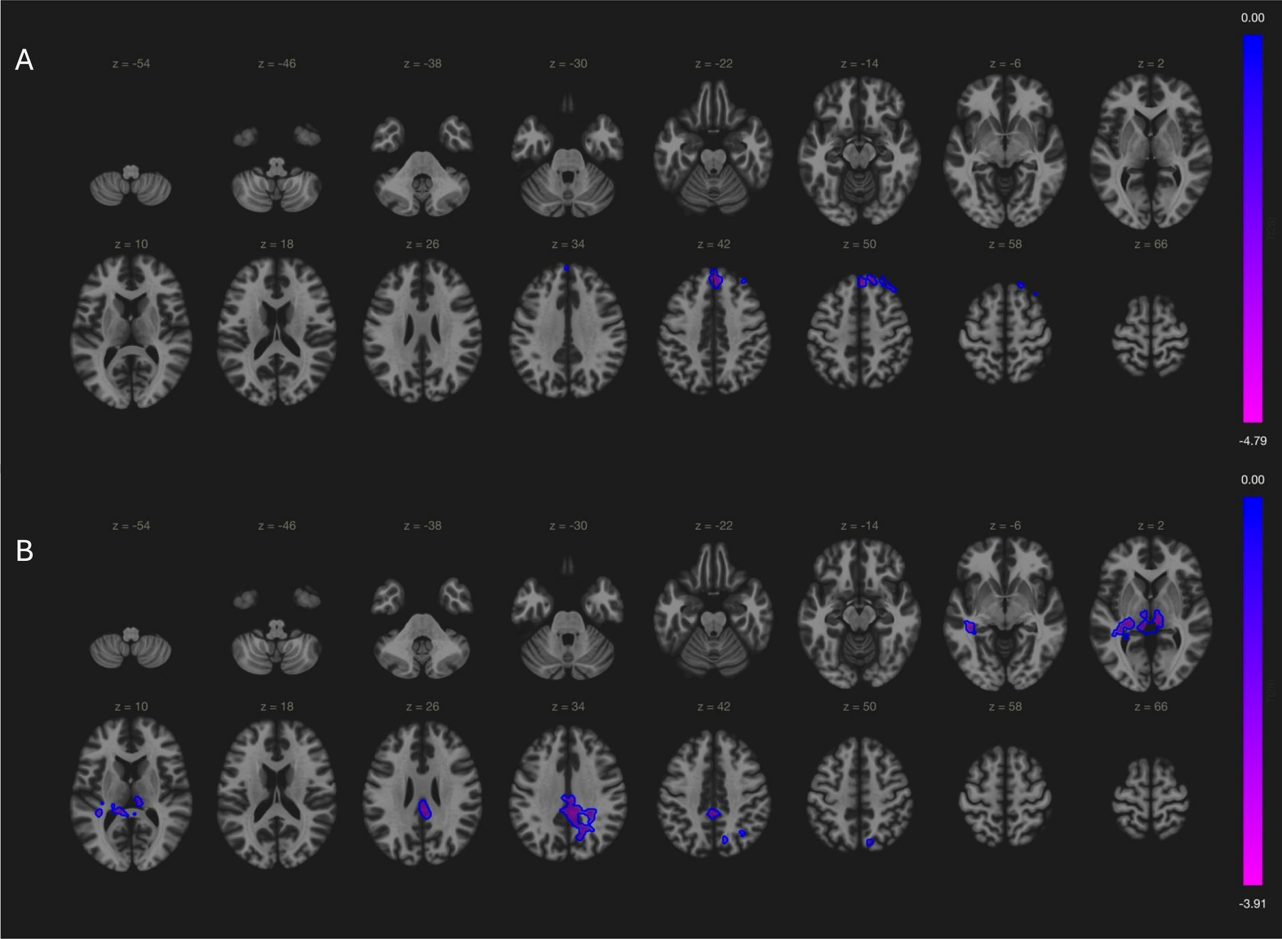
Comparisons of connectivity maps between MSA and PD patients. **(A)** MSA patients had decreased cerebellar connectivity with bilateral superior frontal gyrus; **(B)** MSA patients had less language network activity in bilateral posterior cingulate gyri and bilateral thalamus.

In our local cohort, analysis of variance found significant cross-group differences in cerebellar network (bilateral superior frontal gyrus; (2, 38, 50); cluster size = 112; p-FDR = 0.009) and language network (bilateral posterior cingulate gyri; (2, −34, 32); cluster size = 357; p-FDR = 0.01), but not sensorimotor network and default mode network. Post-hoc comparisons showed significant difference of cerebellar network and language networks between MSA and PD patients. MSA patients had decreased cerebellar connectivity with bilateral superior frontal gyrus [(2, 38, 50); cluster size = 266; p-FDR = 0.001, Figure 2A], and impaired language connectivity with bilateral posterior cingulate gyri [(4, −22, 34); cluster size = 440; p-FDR = 0.001] and bilateral thalamus [(−12, −30, +2) and (+8, −24, +4); cluster size = 241; p-FDR = 0.001; Figure 2B].

In PPMI cohort, PD patients showed increased cerebellar network activation in cerebellar vermis, brain stem and right thalamus; and decreased language network connectivity with bilateral frontal pole, frontal orbital cortex, precuneus and lateral occipital cortex (Supplementary Figure 4).

### 3.4 Correlation between clinical markers and iron deposition

**Figure 3:**
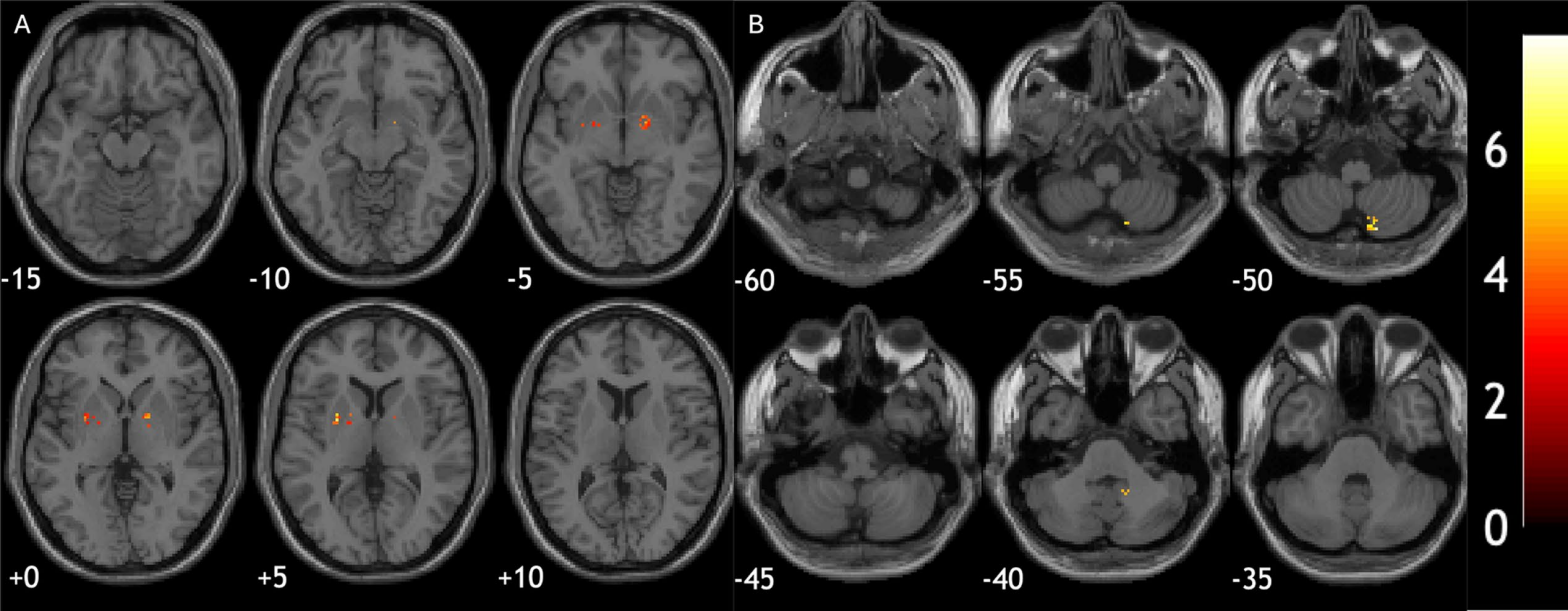
Relationship between brain iron deposition and clinical assessment scores. **(A)** Iron deposition in bilateral putamen correlated with worse motor scores in MSA patients. **(B)** Iron deposition in cerebellum was associated with worse cognitive performance in PD patients.

Iron deposition at ROI level did not correlate with cognition or motor symptoms in 3 diagnostic groups. However, in voxel-wise analysis, quantitative susceptibility in bilateral putamen was correlated with worse UMSARS motor scores in MSA patients (−26, 5, 4; cluster size = 413; p-FDR < 0.001 and 15, 5, −2; cluster size = 479; p-FDR < 0.001, Figure 3A). In PD patients, iron deposition in cerebellum was associated with worse MoCA scores (16, −64, −53; cluster size = 301, p-FDR = 0.01, Figure 3B).

### 3.5 Correlation between clinical markers and brain network

**Figure 4:**
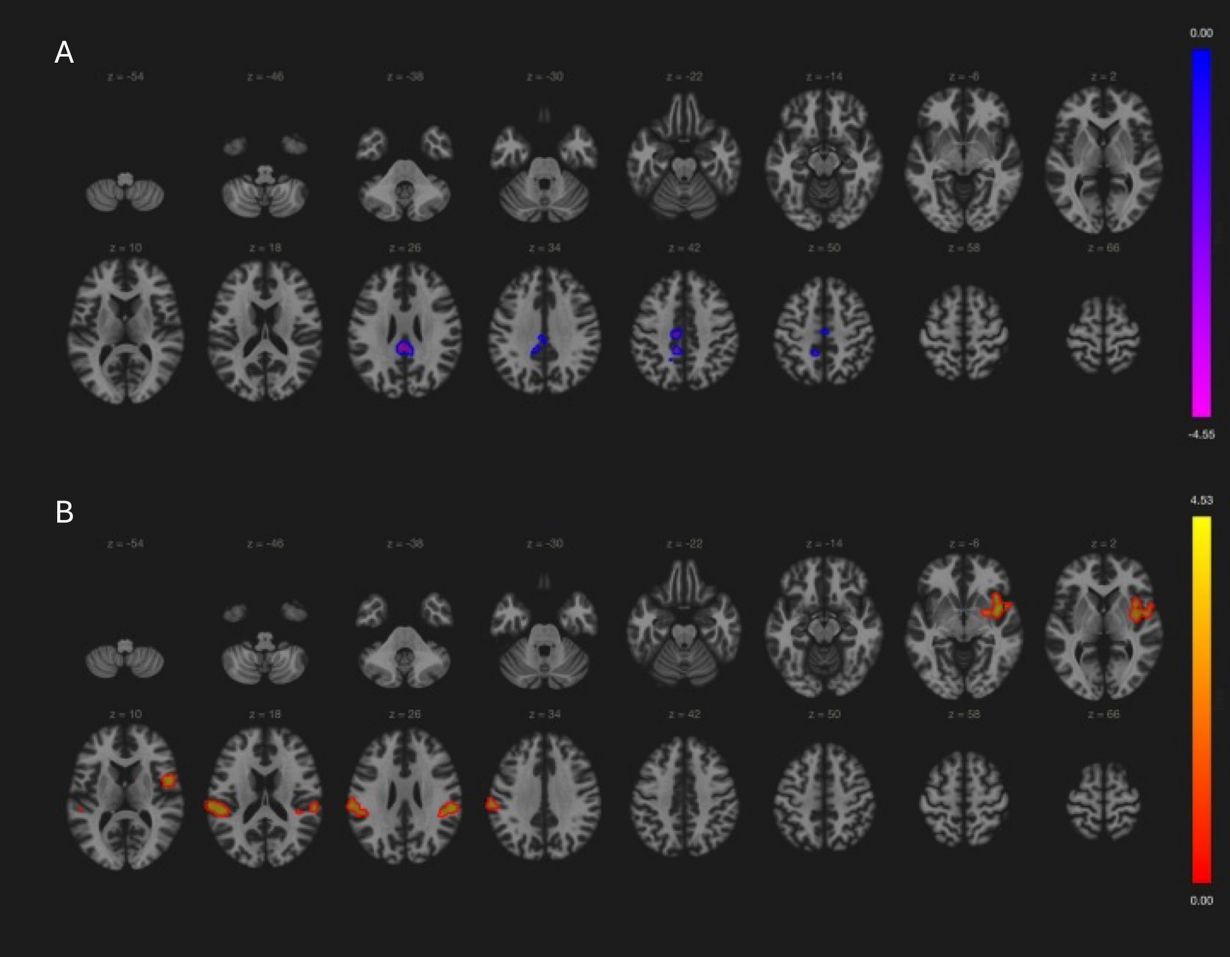
Relationship between clinical assessment scores and brain network in PD patients (PPMI cohort results). **(A)** Cerebellar network connectivity with primary motor and sensory cortices were associated with better (lower) UPDRSm scores (−4, −36, 26; size = 553, p-FDR = 0.03). **(B)** Language network connectivity in left insular cortex (38, 0, −4; size = 402, p-FDR = 0.001), bilateral supramarginal gyri (−62, −24, 22; size = 382, p-FDR = 0.001 and 56, −30, 24, size = 187, FDR = 0.03) had positive correlation with MoCA score.

In local PD cohort, cerebellar network connectivity in cerebellar vermis, brain stem and left thalamus positively correlated with UPDRSm scores, while in PPMI PD patients’ cerebellar connectivity with precentral, posterior central cortices was negatively associated with motor symptoms (Table 2 and Figure 4). Association between cerebellar network and motor symptoms was not significant in MSA and PSP patients. Interestingly, default mode network connectivity was not associated with cognitive performance in the patients. Instead, MoCA scores was related to language network connectivity in PD and PSP patients (Table 2 and Figure 4). Notably, increased involvement of primary motor cortex in language network was negatively related to cognitive performance, while language network activity in bilateral frontal gyri and left angular gyrus was associated with better cognitive scores in those patients.

**Table 2:** Relationship between brain functional network and clinical assessments.

Group	Analysis	Cluster (x, y, z)	Size	Size p-FDR	Anatomical location
PD	Cerebellar network and UPDRSm (positive correlation)	0, −48, 2	142	0.004	Cerebellar vermis
		−4, −32, −8	114	0.008	Brain stem
		−8, −30, 2	92	0.02	Left thalamus
	Language network and MoCA (positive correlation)	14, 2, 58	213	0.02	Right superior frontal gyrus
PSP	Language network and MoCA (positive correlation)	−44, −56, 48	324	<0.001	Left angular gyrus
		38, 8, 42	165	<0.001	Right middle frontal gyrus
		46, 48, 8	142	0.001	Right frontal pole
		−48, 6, 38	110	0.001	Left middle frontal gyrus
		56, 30, 20	74	0.04	Right inferior frontal gyrus
	Language network and MoCA (negative correlation)	−22, 20, 60	110	0.008	Left precentral gyrus
		4, −14, 70	108	0.008	Right precentral gyrus

### 3.6 Relationship between iron deposition and functional networks

In MSA patients, iron deposition in dentate nucleus and putamen was associated with decreased cerebellar network activity within the cerebellum and with thalamus. While quantitative susceptibility in globus pallidum was associated with decreased language network activity in left supramarginal gyrus and cerebellum, and increased language network connectivity with postcentral cortex, precuneus and right frontal pole.

In PD patients, substantia nigra iron deposition was associated with both decreased cerebellar connectivity with thalamus and decreased language network activity in left angular gyrus and cerebellum. In PSP patients, language network connectivity was influenced by iron deposition in substantia nigra and subthalamus nucleus, both causing decreased network activity in left angular gyrus. Interestingly, iron deposition in substantia nigra was also associated with increased precentral gyri activity in language network, which was linked to worse cognitive performance (Table 3).

**Table 3:** Relationship between brain functional network and iron deposition.

Group	Network	Iron deposition	Cluster (x, y, z)	Size	size p-FDR	Anatomical location
MSA	Cerebellar (negative correlation)	Dentate nucleus	20, −48, −30	309	0.04	Right cerebellum
			10, −14, 14	62	0.02	Right thalamus
			−4, −20, 14	49	0.04	Left thalamus
	Cerebellar (negative correlation)	Putamen	−26, −72, −38	258	0.03	Left cerebellum
			8, −46, −38	90	0.04	Right cerebellum
	Language (negative correlation)	Globus pallidus	−40, −38, 30	208	0.02	Left supramarginal Gyrus
			34, −64, −48	170	0.02	Right cerebellum
			−28, −60, −53	155	0.02	Left cerebellum
			−12, −20, −34	154	0.02	Brain stem
	Language (positive correlation)	Globus pallidus	16, −42, 80	164	0.02	Right postcentral gyrus
			14, −54, 24	137	0.03	Precuneus cortex
			12, 66, 0	132	0.03	Right frontal pole
PD	Cerebellar (negative correlation)	Substantia nigra	2, 2, 2	340	0.009	Bilateral thalamus
	Language (negative correlation)	Substantia nigra	−52, −54, 24	676	<0.001	Left supramarginal gyrus and angular gyrus
			−10, −66, −36	379	0.003	Left cerebellum
PSP	Language (negative correlation)	Subthalamus nucleus	−38, 40, 0	311	0.005	Left frontal pole
			36, 44, −2	356	0.005	Right frontal pole
			56, 38, 18	241	0.02	Right middle frontal gyrus
			−44, −56, 44	230	0.02	Left angular gyrus
			60, −48, −6	227	0.02	Right middle temporal gyrus
	Language (negative correlation)	Substantia nigra	10, 30, 32	468	0.008	Right middle frontal gyrus
			−46, −56, 44	309	0.02	Left angular gyrus
	Language (positive correlation)	Substantia nigra	8, − 18, 64	208	0.01	Right precentral gyrus
			−6, −24, 74	162	0.01	Left precentral gyrus

## 4 Discussion

The current study demonstrated disease-specific changes of brain functional network and iron deposition pattern in PD, MSA and PSP patients. Further analyses have shown the relationship between those imaging markers and clinical presentations, as well as an association between iron deposition and brain network changes. While the study is limited by its small sample size, the preliminary findings provide new evidence that iron deposition in movement disorders could be related to brain network dysfunction, hence motor and cognitive symptoms.

Previous studies have illustrated different patterns of brain network disruption in Parkinsonism (Filippi et al., 2019). Compared to healthy individuals, previous evidence suggests decreased basal ganglia-motor cortex connectivity, decreased striatal-midbrain connectivity, increased sensory motor network and cerebellar network activity in PD patients (Tuovinen et al., 2018; Manza et al., 2016). The current study also found increased cerebellar network connectivity within cerebellum and with brain stem and thalamus in PD patients with worse motor performance, which is consistent with previous reports (Kaut et al., 2020; Wang et al., 2023). On the other hand, we also found better cerebellar connectivity with primary cortices was associated with better motor scores, which is a possible compensation mechanism (Wang et al., 2023). MSA patients in the current study has also been shown to have more significant cerebellar and language network disruption compared to PD patients, mostly in line with existing literature (Baggio et al., 2019; Kawabata et al., 2019).

It is also noteworthy that language network, but not default mode network activity was related to cognitive performance in both PD and PSP patients. Our validation analysis with PPMI cohort further confirmed language network, but not DMN impairment in PD patients, and its association with cognitive impairment. DMN disruption has been recognized as a major brain network marker in Alzheimer’s disease and a culprit for cognitive decline (Ibrahim et al., 2021), and a body of literature also suggests its relationship with cognitive decline in movement disorders (Diez-Cirarda et al., 2018; Hou et al., 2017). However, while there is convincing evidence on the involvement of DMN in the development and spreading of AD pathology (Han et al., 2023; Giorgio et al., 2024), such link in movement disorders is yet to be established. On the other hand, the current study suggests that language network might be involved in cognitive changes in patients with Parkinsonism. Neurolinguistic symptoms are most prominent in PSP (Peterson et al., 2021), while PD and MSA patients can also have speech difficulties at later stages (Rohl et al., 2022; Cuoco et al., 2021). Altered language network connectivity has been already noted in MCI patients due to AD (Pistono et al., 2021), and given the fact that language processing is intertwined with other complex cognitive tasks, it could be speculated that its disfunction is accountable for cognitive impairments in patients with movement disorders (Williams et al., 2022; Hertrich et al., 2020). Moreover, a recent report by Cai et al. also directly stressed the importance of language network in PD patients’ cognitive performance (Cai et al., 2024) However, it should be noted that more detailed cognitive assessments on language and other cognitive domains, as opposed to a general MoCA screening test, as well as task-based functional imaging, are needed to further elucidate the specific role of language network in those disorders (Kemik et al., 2024). Further studies on Parkinsonian-specific cognitive impairment related brain network patterns as opposed to cognitive impairments of other etiology will also be helpful. Nevertheless, the relationship between overall cognitive performance and language network could be an interesting clue for further investigations.

Regarding iron deposition, findings from current study falls in line with the literature in that MSA is characterized by iron deposition in putamen while PSP patients have significant iron load in globus pallidum (Wang et al., 2012; Wang et al., 2016). Further, we also observed an association between putamen iron deposition and motor symptoms in MSA patients. Interestingly, cerebellar iron deposition in PD patients was found to be related to worse MoCA score, suggesting a possible link between subcortical iron load and neocortex dysfunction. These observations led the speculation that subcortical dysfunction related to iron deposition might cause brain abnormalities via brain network, which connects the distal regions structurally and functionally.

With the prior hypothesis in mind, we further examined whether magnetic susceptibility in disease-specific regions is associated with network changes. Iron deposition was associated primarily with reduced cerebellar network connectivity within the cerebellum and with basal ganglia. Language network, on the other hand, seem to show complex changes related to iron deposition. In particular, activity in key language hubs including left angular gyrus, left supramarginal gyrus and middle frontal gyrus (Fedorenko et al., 2024), was negatively influenced by iron deposition. While there seemed to be a shift toward more primary cortices’ involvement in language network, such as precentral and postcentral gyri, corresponding to higher iron load in those subcortical nuclei. The latter might reflect a compensatory mechanism, albeit possibly ineffective, as increased precentral and postcentral gyri’s involvement in language network was shown to be associated with worse MoCA score in prior analysis. These findings are supported by recent report that altered STN-language network connectivity is related to speech-related cognitive performance (Cai et al., 2024).

The pathological mechanism of iron deposition’s effect on brain connectivity could be multifaceted. Previous studies have shown iron overload could directly cause neuronal ferroptosis and neurodegeneration in PD (Mahoney-Sanchez et al., 2021). Further, iron deposition could also cause oxidative stress and neuroinflammation, facilitating neurodegeneration (Levi et al., 2024). In the context of Parkinson’s disease, iron overload’s neurotoxicity is further potentiated by its interaction with α-synuclein oligomers (Deas et al., 2016). Moreover, a substantial body of literature has established the close link between α-synuclienopathy and iron. In addition to inducing conformation change of α-synuclein, iron is believed to be involved in both post-transcriptional and post-translational regulation of α-synuclein, as well as disrupting its degradation (Chen et al., 2019). Interestingly, however, there has also been reports that brain iron enrichment is able to confine network spreading of α-synuclein (Dauer et al., 2021). Considering iron deposition’s negative effect on brain connectivity, it might be posited that by cutting off communications between distal neurons, iron deposition and its subsequent cascade helps to confine the pathology at the cost of local neuronal damage. On the other hand, given subcortical iron deposition’s effect on neocortical networks, how the changes in subcortical circuits, which are often described as oscillating loops, modulate neocortical brain networks also needs to be elucidated (Singh, 2018).

The current study is first limited by the small sample size. While we were able to demonstrate established network findings and documented susceptibility changes in our patients, exploratory findings must still be considered with caution and further validation is required. Secondly, the current study cohort consisted of patients only, and inclusion of healthy volunteers will be much needed to further elucidate disease-specific changes in relation to healthy individuals. Lastly, ICA based network segregation was applied in the current analysis, as the data-driven approach offered less biased network target selection. However, alignment with other studies’ fundings is complicated and more sophisticated networks, such as pathways between basal ganglia neocortex and did not emerge in current analysis—probably due to limitations of sample size and temporal resolution of fMRI. Further studies need to take careful methodological considerations, such as whether more refined seed-based analysis is preferred.

Despite the above limitations, the current study is among few studies that reported the association between iron deposition and network dysfunction in Parkinsonian disorders. The findings have also placed emphasis on language network disruption in movement disorders, and we hope they may serve as initial clue for further investigations.

## Data Availability

PPMI data used in the preparation of this article was obtained on [2024-11-25] from the Parkinson’s Progression Markers Initiative (PPMI) database (https://www.ppmi-info.org/access-data-specimens/download-data), RRID:SCR_006431. For up-to-date information on the study, visit https://www.ppmi-info.org.
