# Supplementary material for: Iron deposition is associated with motor and non-motor network breakdown in parkinsonism
Source: Front Aging Neurosci. 2025 Jan 20;16:1518155. doi: 10.3389/fnagi.2024.1518155 (PMC11788357; doi:10.3389/fnagi.2024.1518155)
Supplement: Supplementary file 2 [file Supplementary_file_1.docx]

Supplementary materials

Figure 1. group ICA results


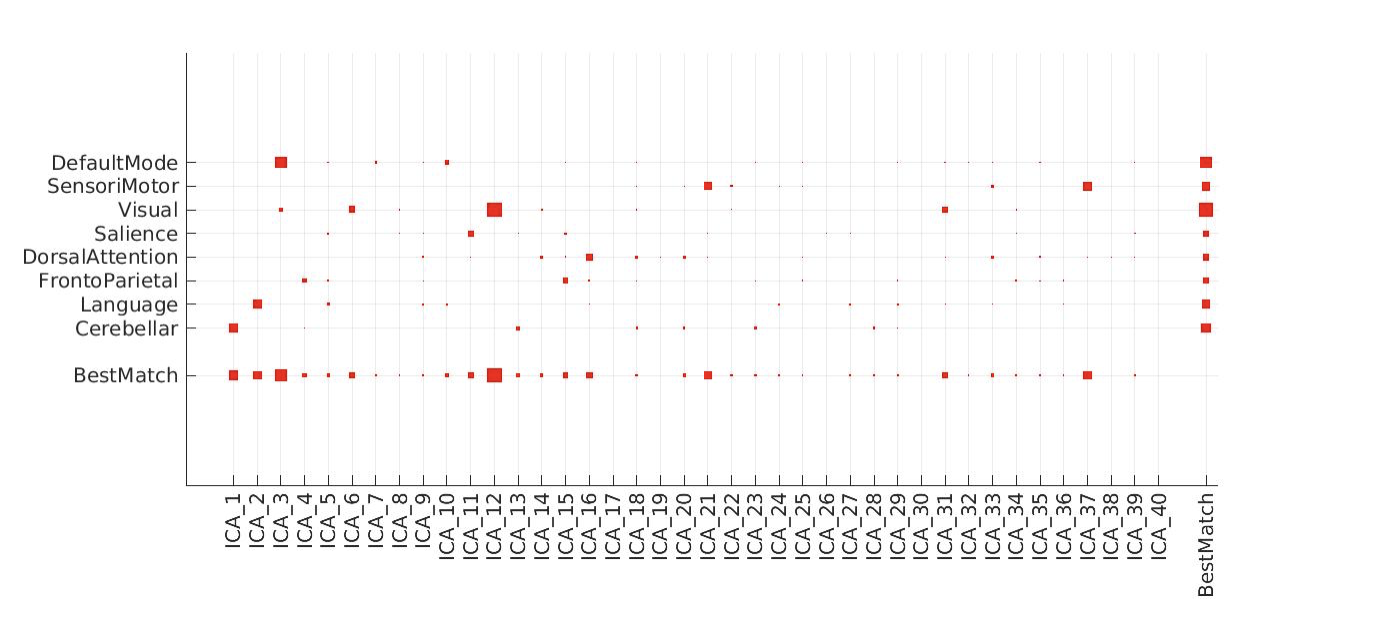


Figure 2. Selected networks of interest


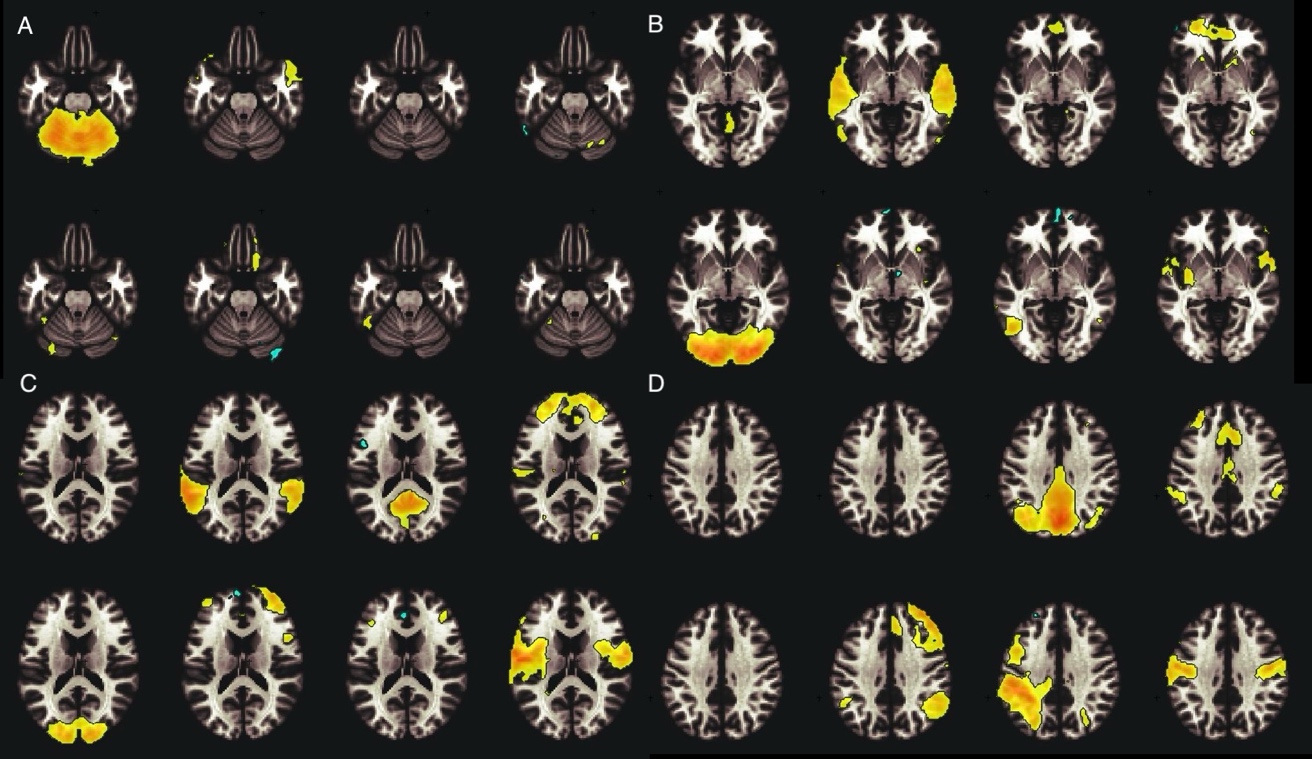


A. Cerebellar network; B. Language network; C. Sensorimotor network; D. Default mode network

Figure 3. Analysis diagram


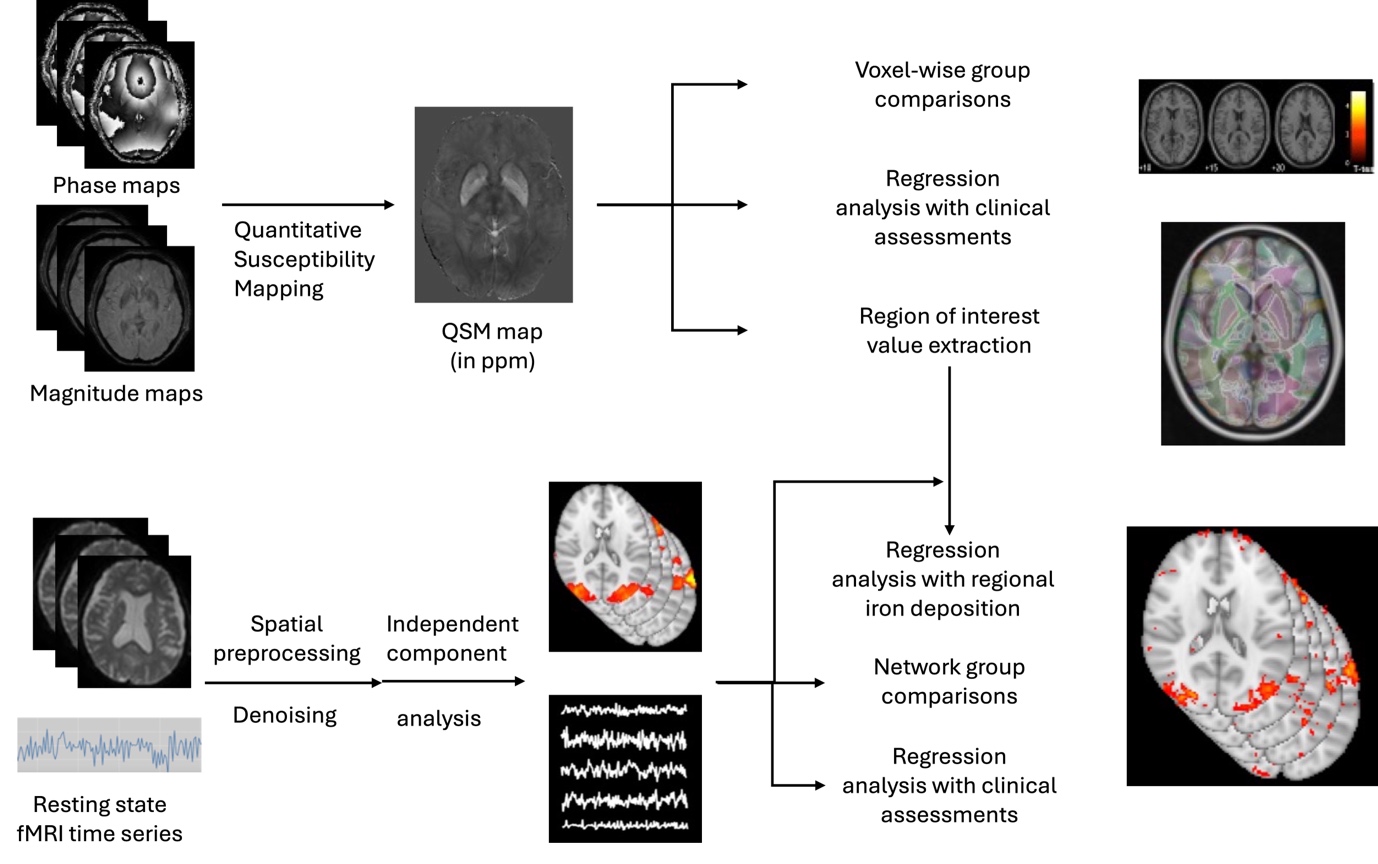


Figure 4. Functional network comparisons between PD and HC from PPMI cohort


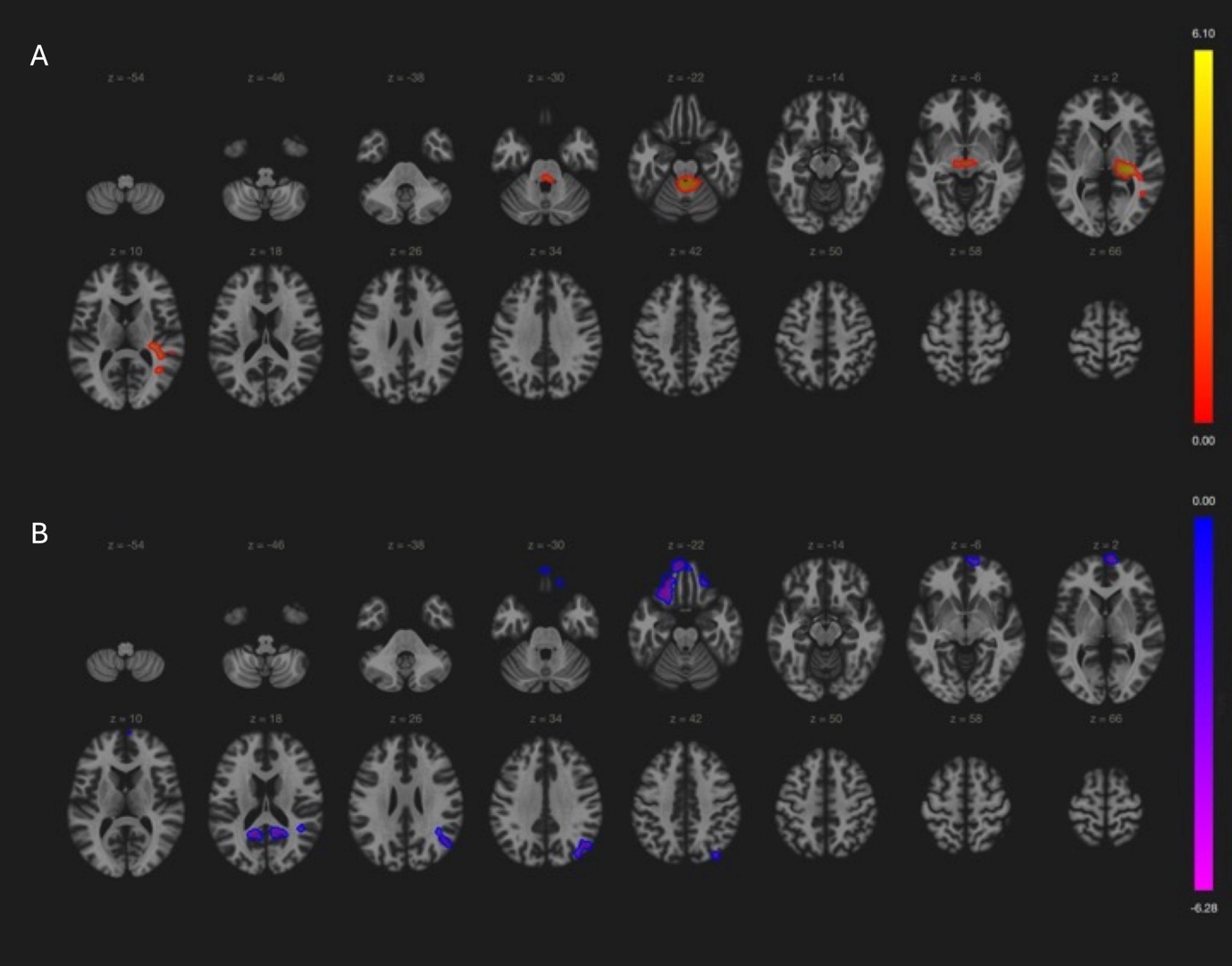


1. Increase cerebellar network connectivity in PD patients; B. Decreased language network connectivity in PD patients.
